# A Reference Architecture for Cloud–Edge Meta-Operating Systems Enabling Cross-Domain, Data-Intensive, ML-Assisted Applications: Architectural Overview and Key Concepts

**DOI:** 10.3390/s22229003

**Published:** 2022-11-21

**Authors:** Panagiotis Trakadas, Xavi Masip-Bruin, Federico M. Facca, Sotirios T. Spantideas, Anastasios E. Giannopoulos, Nikolaos C. Kapsalis, Rui Martins, Enrica Bosani, Joan Ramon, Raül González Prats, George Ntroulias, Dimitrios V. Lyridis

**Affiliations:** 1 National and Kapodistrian, Department of Port Management & Shipping, University of Athens, Psachna, Evia, 34400 Athens, Greece; 2 CRAAX, Universitat Politecnica de Catalunya, 08800 Vilanova i la Geltru, Spain; 3 Martel Lab, Martel GMBH, 6900 Lugano, Switzerland; 4 Four Dot Infinity PC, Leof. Kifisias 208, 15231 Chalandri, Greece; 5 Smart Energy Lab, Avenida 24 de Julho, n° 12, 1249-300 Lisboa, Portugal; 6 Whirlpool EMEA, Via Carlo Pisacane n. 1, 20016 Pero, Italy; 7 IDNEO Technologies SAU Nextium, Carrer Rec de Dalt, 3 Mollet del Vallès, 08100 Barcelona, Spain; 8 Retevision I SA Cellnex, Avinguda del Parc Logistic, 08040 Barcelona, Spain; 9 Hydrus Engineering SA, Leof. Mesogeion 515, 15343 Agia Paraskevi, Greece; 10 Laboratory for Maritime Transport 9, School of Naval Architecture and Marine Engineering, National Technical University of Athens, Heroon Polytechniou St, Zografou, 15773 Athens, Greece

**Keywords:** edge computing, reference architecture, meta-operating system, decentralized intelligence, IoT–edge–cloud continuum, federated learning, swarm learning

## Abstract

Future data-intensive intelligent applications are required to traverse across the cloud-to-edge-to-IoT continuum, where cloud and edge resources elegantly coordinate, alongside sensor networks and data. However, current technical solutions can only partially handle the data outburst associated with the IoT proliferation experienced in recent years, mainly due to their hierarchical architectures. In this context, this paper presents a reference architecture of a meta-operating system (RAMOS), targeted to enable a dynamic, distributed and trusted continuum which will be capable of facilitating the next-generation smart applications at the edge. RAMOS is domain-agnostic, capable of supporting heterogeneous devices in various network environments. Furthermore, the proposed architecture possesses the ability to place the data at the origin in a secure and trusted manner. Based on a layered structure, the building blocks of RAMOS are thoroughly described, and the interconnection and coordination between them is fully presented. Furthermore, illustration of how the proposed reference architecture and its characteristics could fit in potential key industrial and societal applications, which in the future will require more power at the edge, is provided in five practical scenarios, focusing on the distributed intelligence and privacy preservation principles promoted by RAMOS, as well as the concept of environmental footprint minimization. Finally, the business potential of an open edge ecosystem and the societal impacts of climate net neutrality are also illustrated.

## 1. Introduction

Over the last years, the expeditious spread of Internet of Things (IoT) technology has opened numerous opportunities to develop intelligent and big data applications, fueled by recent technological advances in the fields of machine learning (ML) [1] and cloud computing [2,3,4]. These data-intensive applications are required to operate across the cloud-to-edge continuum, where cloud, edge, core network, radio-access network, sensors and data itself coexist and collaborate [5,6,7]. While today numerous proprietary and open source technical tools support IoT-to-edge-to-cloud scenarios as a single commodity, solutions that are able to truly bring computation and intelligence closer to the edge nodes, where the data are generated, are still facing several technical, societal and business barriers [8]. The main complication regarding this transition is that current solutions solely focus on the hierarchical orchestration of resources, services and data, necessitating data migration from IoT-to-edge-to-cloud, and vice versa [9,10,11,12]. This hierarchical architectural model inevitably results in predefined topologies that exhibit several limitations (see Section 2), thus becoming the main blocking point in implementing the next generation of edge intelligence applications. Critically, to support volatile applications across the IoT–edge–cloud continuum that also enable cognitive decisions in heterogeneous environments, it is required that the computational resources of all parts of the continuum can be rapidly reconfigured in a dynamic manner, while at the same time preserving their operational performance.

Meta-operating systems (meta-OSs) are not a novel concept. In principle, a meta-OS is built on top of a typical operating system, providing basic OS functionalities (hardware abstraction, low-level device control, etc.), allowing the communication between different nodes and managing the resources across multiple computers [13]. Recently, sectors such as robotics have shown a proliferation of such a meta-OS approach, with the robot operating system (ROS-2) being one of the most popular [14,15]. In addition, meta-OSs developed in various domains enable different levels of coordination among heterogeneous agents/devices [13,16]. Notably, the multi-agent collaboration enables swarm intelligence, which refers to the capacity of the swarm (or multiple agents) to complete complex tasks that are not achievable by single entities, without relying on the existence of a central “brain” [17,18]. In this context, a meta-OS can support distributed applications across the IoT–edge–cloud continuum, enabling decentralized, federated and/or swarm intelligence at the far-edge.

Notwithstanding the foregoing, the practical establishment of the meta-OS comes with some key challenges. Firstly, it is estimated that by 2023, more than 30 billion devices will have been connected to the Internet [19]. These devices are built upon a wide diversity of hardware platforms, operating systems and communication protocols, posing an extreme complexity in integrating their computational resources within the IoT–edge–cloud continuum. While current architectural solutions support integration of CPU-based devices, they lack unified abstraction schemes for the micro-controller unit (MCU) devices. In addition, a meta-OS must support scalability and resilience of orchestration in the computing continuum [20]. Once the resources from the CPU and MCU-based devices become available, orchestration mechanisms must be applied. Current orchestration schemes become cumbersome and error-prone, and thus they will not be able to function in dynamic, highly volatile, heterogeneous and hyper-distributed environments foreseen in the future edge intelligence applications. Hence, the realization of novel schemes for decentralized peer-to-peer coordination is also required [21,22]. Furthermore, the hierarchical architectural framework has a significant impact on the data offloading mechanism and the related network parameters. Within the period 2016–2021, there have been 850 ZB of data generated by mobile users and IoT devices [19]. In the existing IoT–edge–cloud schemes, where data is transferred across the hierarchical continuum, the network cannot scale with the same speed of data growth, primarily attributed to the required overhead that is needed for transferring the data from the IoT to cloud layer (i.e., vertical communication). This has a direct impact on latency and throughput, which, in turn, hinders the performance of real-time applications. Additionally, in the current hierarchical schemes, data is required to travel across multiple public and private cloud infrastructures, often disrespecting privacy preservation and increasing the cybersecurity attack surface [23,24,25]. Finally, the rapid proliferation of cloud–edge applications also involves the uncontrollable inflation of the associated carbon footprint [26,27]. Current data distribution from edge to cloud significantly impacts the ICT carbon footprint, whereas future meta-OSs need to move towards greener and sustainable solutions, boosting the concept of climate net neutrality.

In this work, we propose a reference implementation of a meta-OS, targeting the transformation of the current vertically-siloed cloud–edge–IoT architectures into a dynamic, distributed and trusted continuum, capable of facilitating next-generation applications. This reference architecture of a meta-operating system (RAMOS) can span across heterogeneous devices and capacities to fulfil the requirements of the next-generation applications in such a way that data volume, variety, interoperability, and velocity are handled efficiently and securely. This architectural transformation inherently implies the shift of the ML approaches adopted in the computing continuum from central learning to distributed learning, enabling decentralized intelligence at the far edge. To this end, the peer-to-peer architecture supported by RAMOS intrinsically points towards federated learning (FL) and swarm learning (SL) where data and ML models remain at the edge rather than being centralized. Following the technological trends for automation and network intelligence, RAMOS can also support ML operations via a dedicated layer [28,29,30].

The rest of the paper is organized as follows: Section 2 illustrates the motivation behind the present work and the requirements of the meta-OS, while the overall contribution at the architectural, data management and decentralized intelligence levels is outlined in Section 3. More detailed description of the proposed meta-OS reference architecture is fully provided in Section 4, whereas potential adoption of RAMOS-based schemes is demonstrated in Section 5, considering five real-world scenarios. Finally, Section 6 presents the potential business and societal impacts due to the adoption of RAMOS, while Section 7 concludes the paper.

## 2. Motivation and Meta-OS Requirements

The motivation of this work originates from the need to describe the specifications of a meta-OS architecture which can enable the integration of novel ML-driven applications and services [31,32]. The following list summarizes the requirements for a reference meta-OS architecture that will enable a dynamic, distributed and trusted IoT–edge–cloud continuum and describes why current solutions are not adequate to conform to these challenges:

- *Provide an effective management of a wide diversity of hardware platforms, operating systems and communication protocols.* While in the core cloud the variety of hardware, operating systems and communication protocols is today largely tackled and abstracted by solutions such as Kubernetes [33], the variety in the computing continuum ranges from hardware platforms based on ARM processor architectures to 8-bit microcontrollers, supporting different communication protocols (such as LORA, WIFI, BLE, etc.), and being built on top of different operating systems (Android, iOS, ROS, RIOT, Yocto, Zephyr, etc.) [34]. Evidently, this heterogeneity in both hardware and operating software cannot be managed using conventional architectures. To support the seamless management of such a diversity of devices, a unifying abstraction approach is required to both manage and monitor such resources, as well as to deploy services “as functions” on top of them. The required architectural transformation should build on best innovations offered by *cloud-native* solutions and making them *edge-native*.
- *Support the scalability and resilience of orchestration in the computing continuum*. Current state-of-the-art (SOTA) solutions to IoT–edge–cloud orchestration of resources and services are cloud-centric and leverage predefined resource providers [34,35]. This has a significant impact on (i) *scalability*: the latency of orchestration directly increases with the number of devices to be managed and their (network) distance from the orchestrator, and the infrastructure can scale only within a predefined pool of resources; (ii) *resilience*: partial availability of the connectivity may affect the ability of the orchestrator to recover and ensure the end-to-end functionality of the cloud–edge service, and when the predefined pool of resources cannot cope with system load or failures, no alternative is available. In this context, current hierarchical approaches partially mitigate this issue. To tackle this problem effectively, novel solutions are needed to either *move the gravity of decision at the edge* or at least empower the edge to take part of the decisions and/or predictions, thus *decentralizing the systems orchestration and enabling peer-to-peer coordination*, including the dynamic discovery and inclusion of new resource providers.
- *Minimize the offloading of current vertically-siloed architectures and its impact on the network*. The enormous number of devices joining the continuum irrevocably implies that vast amounts of data are generated at the edge, increasing the challenges on the data processing layer and creating bottlenecks, especially in the execution of heavy ML-based tasks. Vertical offloading patterns, commonly used in cloud–edge and centralized learning approaches, heavily rely on low network latency and considerable bandwidth capacity [36,37]. This unfavorable dependency can be addressed by adopting a peer-to-peer offloading strategy instead of a hierarchical architecture. However, while on the one side, such peer-to-peer offloading and moving computation at the edge may reduce the usage of the network to transport data, on the other, it requires that services and applications are the ones that move towards the data. To this end, such challenges require putting data at the center of orchestration (introducing the concept of *data-gravity*), where data remain at the edge, a fact that leads to reduced network usage and associated ML models footprint.
- *Increase security and privacy-awareness of ML distributed applications*. While data increasingly play a central role in the development of innovative connected apps and services, concerns of end-users and organizations with respect to data sharing, data ownership and privacy preservation, slow down the take-up of ML methods and limit the access to data economy to a remarkable set of global players [38,39,40]. In a distributed system, ensuring data ownership and privacy preservation becomes even more complex. To increase the trust toward data sharing and to support the growth of ML approaches, solutions that *increase data owners’ control and ensure privacy preservation* in the computing continuum are required. In contrast to server-centric existing solutions for data sharing, privacy-preserving verifiable data sharing systems that are based on blockchain can be adopted for the peer-to-peer RAMOS architecture [41,42], while also conforming to the decentralized nature of the IoT–edge–cloud continuum.
- *Measure and reduce carbon footprint of cloud–edge applications*. While clear mechanisms and standards have been developed to measure and handle the energy efficiency of the data centers and their carbon footprint, SOTA methods are far from achieving that in the computing continuum [26,27,43]. Although energy consumption is easy to measure in an owned datacenter, cloud providers do not grant access to such information and, consequently, knowing or simply estimating the network related consumption from cloud-to-edge is even more challenging. Without measurements or models to estimate them, taking decisions aimed at reducing the carbon footprint of cloud–edge applications is infeasible. In this context, techniques, policies and standard APIs to make available energy-related information are a key requirement to enable appropriate *energy optimization* of ML applications in the computing continuum. Furthermore, additional techniques to enhance the energy-efficiency of the individual devices and resources in the IoT–edge–cloud continuum shall be adopted, focusing on various stages of the data transfer and processing chain. For instance, local data pre-processing before offloading to an edge server can significantly reduce the energy associated with the data transmission at the cost of lower result accuracy [44], different data communication methods can be utilized by the devices depending on the data transmission queue, estimated network conditions and the device moving speed [45], and finally, merging of several keep-alive connections into one can be realized to considerably reduce the energy consumption [46].

## 3. Proposed Reference Meta-OS Architecture and Functionalities

To tackle the aforementioned challenges and requirements, the presented meta-OS reference architecture shall enable the creation of a peer-to-peer continuum for IoT applications, spanning heterogeneous software and hardware architectures and fostering the realization of a decentralized intelligence. This transformational shift will further allow migration from the current 80–20% data balance between cloud and edge to a 20–80% balance [47,48,49]. This rebalance will disrupt the current business models around data sharing by returning control to data producers, while, at the same time, providing new means to control and reduce the carbon footprint of IT services.

To enable the shift from the hierarchical continuum to peer-to-peer continuum and from static to dynamic management of infrastructures, the coordination across cloud nodes is essential. In the proposed meta-OS architecture, the coordination of different nodes:

- Enables the exchange of information about available resources
- Allocates resources in a dynamic and optimized manner
- Utilizes resources to deliver services and applications.

**Figure 1:**
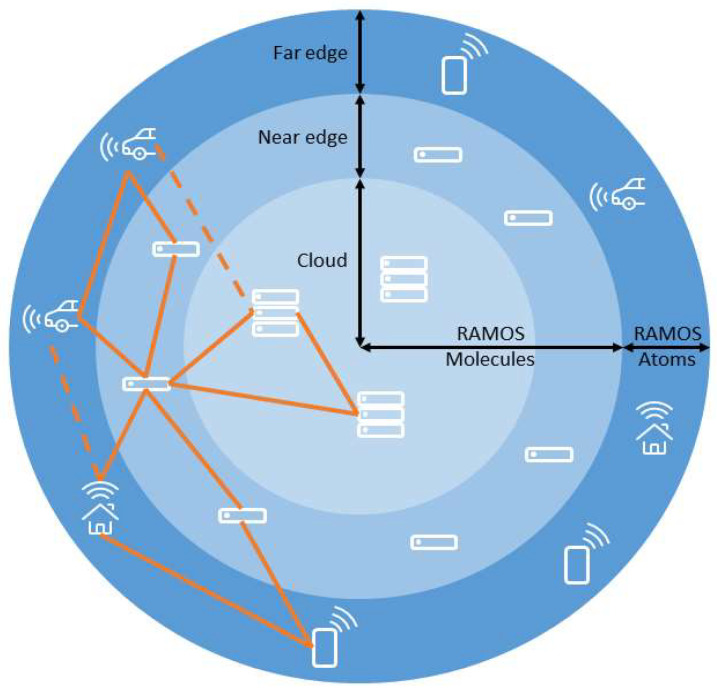
Constellation of RAMOS nodes towards a peer-to-peer continuum.

The coordination, and hence the links to enable the creation of decentralized applications, occurs dynamically, taking into account available resources (i.e., in terms of capacity and reachability, in case of intermittent connectivity). A node, in this sense, can span from a cloud computing cluster (such as a Kubernetes cluster) to an embedded device (such as a robot in a factory). Of course, given this definition, not all nodes can offer the same capacities (Figure 1): the smallest ones (named RAMOS “*Atoms*”) are very simple and provide only a subset of the meta-OS services, requiring coordination and support by more capable nodes (RAMOS “*Molecules*”, consisting of several “*Atoms*”).

**Figure 2:**
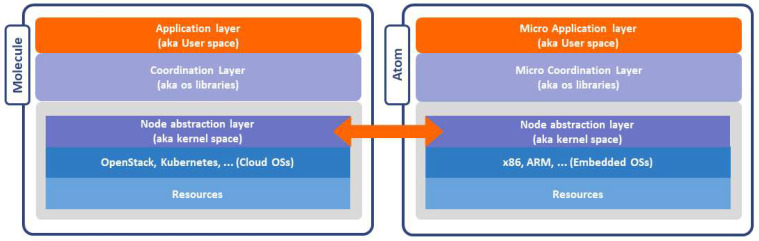
High-level meta-operating system architecture.

In RAMOS architecture, a resource can also be quite heterogeneous: it could be a service, a data set, a hardware capacity or a sensor. Figure 2 shows the high level meta-OS architecture: (i) resources of each node (blue) are abstracted and advertised via a peer-to-peer protocol thanks to the node abstraction layer (deep purple—the *meta-OS kernel space*, managing meta-processes); (ii) the coordination layer (light purple—the *meta-OS libraries*) provides basic functionalities to enact cloud–edge processes; (iii) the application layer (orange—the *meta-OS user space*) hosts user services and applications that leverage functionalities exposed by the coordination layer.

As aforementioned, the proposed RAMOS architecture includes two types of nodes: *Atoms* and *Molecules* that coordinate one or several Atoms, as shown in Figure 2. The resources offered by Atoms or Molecules can be categorized and abstracted, taking into account aspects such as type (computation, storage, energy, data, etc.), capacity (available storage, bandwidth, processing) and location. In this context, the computational resources are abstracted in the RAMOS architecture to embrace their heterogeneity, thus promoting a unifying approach to IoT–edge–cloud resource management, where Atoms mainly represent the embedded devices that can run functions and Molecules represent servers and clouds that coordinate Atoms, offering services in the IoT–edge–cloud continuum. While Atoms and Molecules share a wide range of functionalities, their implementations take into account their different capabilities, characteristics and roles in digitalizing physical systems.

**Figure 3:**
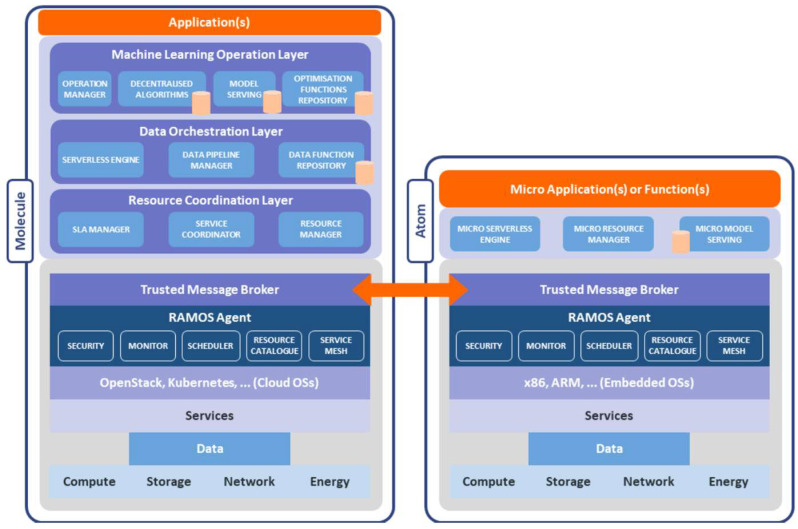
Detailed layered architecture of Atoms and Molecules at component-level.

The overall RAMOS architecture, depicted in Figure 3, follows the fundamental principles of microservices architectures and pushes the concept even further by targeting nanoservices, the simplest form of functions defined in the meta-OS cloud–edge ecosystem executed at the level of Atoms. The separate components of RAMOS architecture are fully described in the following subsections.

### 3.1. Trusted Communication and Collaboration

The core element enabling the creation of a dynamic resources ecosystem spanning across different nodes, is ensured by a decentralized and trust-enabling Message Broker. The broker enables the dynamic establishment of decentralized topologies, thus avoiding single points of failure, while in parallel supporting fault tolerance and high availability, coupled with a reliable message delivery mechanism. Beyond that, the broker is enabling the trustworthiness among nodes; only trusted nodes will be able to take part in the topology. Furthermore, the broker not only allows the communication between the nodes (Atoms or Molecules), between services in the coordination layer, and between services within user applications, but, most importantly, enables the communication between resources exposed by nodes and the coordination layer. Finally, the broker supports both message-based and event-based paradigms, thus enabling time-triggered and event-triggered ΙοΤ applications.

### 3.2. Node and Resource Abstraction through Agents

In order to manage heterogeneous nodes (e.g., Kubernetes clusters, embedded devices), an abstraction layer is a key element. In our envisioned architecture, this abstraction layer is provided by the *RAMOS Agents*. The agents expose resources (computing, storage, network and energy capacities, services, data sources, and sensors), and advertise them in the *Resource Catalogue*, thus enabling their discovery. The catalogue will allow resources to advertise their presence in a seamless and timely manner, copying with heterogeneity, mobility, volatility, and intermittent connectivity characteristics of the edge nodes, while the *Scheduler* allows resources to be allocated and released from a task as needed. The *Monitor* service exposes the status of resources and running tasks, along with their energy expenditure, and thus enables swarm intelligence; nearby nodes (even without a request to execute a task) can detect that another node is unavailable or stopped executing a critical task, and hence take over this task execution. Leveraging the functionality of the previously described components, the *Service Mesh* controller enables dynamic and secure data services chaining, based on simple or more sophisticated mesh algorithms. The dynamic chaining will be supported by means of abstract endpoints, and the service mesh controller, as a sort of clever DNS, converts abstract endpoints into actual endpoints using different information, such as latency and availability. Regarding data service-security, the mesh controller can support self-aware data security by attaching access policies to data and enforcing such policies along the service functions chain. Finally, *Security* agents may identify and mitigate attacks without sending data to the cloud. The cybersecurity agents can be installed in the far edge and will function separately, consuming only the necessary computing power to handle security incidents and ensuring strong protection against malicious incidents.

### 3.3. Resource Coordination

This layer is responsible for discovering the resources needed to comply with a given task (*Resource Manager*). Once the resources are discovered, they are categorized and clustered, a fact that facilitates their optimal use by the service coordinator. The Resource Manager can also be available in Atoms, as the smallest intelligent functionality part of the RAMOS architecture, in order to enable swarm intelligence, based on the ability of self-assigning pending tasks. The *Service Coordinator* uses artificial intelligence (AI) to define the best strategy to coordinate the instantiation of services: spanning from a loosely coupled coordination where Atoms self-allocate service instantiation, to a strong coordination (i.e., orchestration) where Atoms are assigned service instantiation. The Service Coordinator computes and enacts the strategies by: (i) forecasting the resources needed to run a microservice (in terms of sizing, data, location, ownership, context, etc.); (ii) advertising requests for a given set of resources and the need for executing a microservice/task leveraging them; (iii) assigning different microservices/tasks to nodes (while self-assignment of local and external requests is provided by the resource manager). Finally, the *SLA Manager* combines different measurements (such as latency, energy footprint, and throughput), that define the end-to-end performance of an application, to provide a global understanding of the application behavior in the continuum, and trigger the needed recommendations or adaptations to comply with developers defined KPIs. The SLA Manager will prioritize a data-centric approach, thus monitoring not only services, but also data, hence contributing to trigger horizontal and vertical offloading.

### 3.4. Data Orchestration

To promote the shift from service-gravity to data-gravity, applications need to move from service-centric orchestration to data-centric orchestration. This layer provides a set of functionalities to enable data-centric orchestration: a *Serverless Engine*, aimed at deploying data-centric functions, will support advanced NFRs, such as locality, predictability, statefullness and improved composability of functions into pipelines/workflows by referring to the abstract endpoints as managed by the service mesh controller. These properties are crucial for both developing policy-based pipelines in mixed cloud environments, such as fog/edge, hybrid and multi-cloud, as well as making serverless functions more suitable for efficient serving and training of ML models for pipelines. The Serverless Engine, with reduced capacities, will be also available for Atoms, thus homogenizing the functional programming of edge devices and the serverless computing in the cloud. Moreover, a *Catalogue of data-centric functions* will include functions to import, query and sanitize data from heterogeneous data sources, supporting the self-annotation of meta-data (e.g., type, ownership, permissions and privacy constraints), and the self-advertisement of data sources in the resource catalogue, while also being compliant with standardized approaches (e.g., Gaia-X ecosystem which is a federated and secure data infrastructure, whereby data is shared and made available in a trustworthy environment) from the data space ecosystem. Finally, the *Data Pipeline Manager* enables the design of data pipelines by chaining data functions over data sources and by instantiating them via the serverless engine framework (when required, also microservices/functions already deployed and exposed as resources will be chainable). This will allow developers to define intents over functions and pipelines so that the underlying meta-OS functionalities will thrive to satisfy them, as well as to deploy ML models in the edge-continuum, and to support already running models by exposing an API that will allow services to modify parameters of the pipeline and the configuration of the functions.

### 3.5. Machine Learning Operations

This layer aims to offer a set of services to support continuous operation of ML algorithms and models across RAMOS Molecules and Atoms required to truly enable decentralized intelligence at the edge. *The ML Operation Manager* includes a set of tools to manage the training models and to define logics for continuous optimization and adaptation of models across the continuum. In particular, this service will leverage a framework of algorithms to be designed for FL and SL concepts. Due to the specific requirements of edge computing decentralization and local privacy protection, it is required that ML methods are not only distributed across the different RAMOS nodes, but also adapted to the specific characteristics of each node. Therefore, the *Decentralized Algorithms repository* can integrate existing up-to-date approaches of distributed learning. As the training will be carried out on local devices, no private data will leave any of these devices. Only information which is sufficiently aggregated, noisy or appropriately encrypted will be exchanged. Clearly, in order to pursue service minification, a key pillar is to reduce the size of models. For this purpose, a framework of *Machine Learning Optimization Functions* is provided in this layer. For instance, an ML model compression function can provide coding mechanisms, where ML models, model updates and other exchanged information, all of which requiring a huge amount of space in uncompressed form, will be reduced to a fraction of their original size [50]. Other optimization functions that can, in principle be included involve the hyperparameter attunement for the fine-tuning of the learning parameters during the training phase of ML algorithms, and an over-the-air computation (AirComp) method for efficient spectrum utilization and reduction of the energy associated with data transmission from IoT devices [51]. In addition, specialized methods, e.g., structured pruning, can be also be incorporated for federated use case scenarios [52]. Finally, a *Model Serving* service will publish models and version them for an application, either globally (Molecules) or locally where a specific model is computed (Atoms).

## 4. Contributions of RAMOS Architecture

As aforementioned, the presented reference architecture of a meta-OS pursues a number of paradigm shifts aiming at enabling decentralized intelligent applications that will permeate the Internet in the future:

Firstly, at the *architectural level*, the RAMOS moves beyond SOTA in the following directions: (i) discard the vertical (hierarchical) offloading where the model and data are coupled as a single entity and must be migrated to a computationally higher node. This horizontally-oriented architectural change aims to deliver a more fine-grained offloading logic, allowing each node or service to offload a portion of its computational burden, thus reducing the overall data transfer; (ii) support the computing continuum carbon-aware scheduling in the cloud-native ecosystem; (iii) address the shortcomings of current solutions on the discovery of fog/edge devices and the integration of the resources of MCU devices providing a standards-compliant solution that supports location- and context-awareness; (iv) tackle the fallbacks of current SLA (service-level agreement) tools, by implementing an automatic, CNCF-driven (cloud native computing foundation) SLA management; (iv) provide a cybersecurity framework based on the principles of decentralized intelligence to harden intelligent applications [53].

Secondly, at the *data management level*, the proposed architecture: (i) promotes data sharing in a privacy-preserving manner by addressing the problem of statistical heterogeneity and non-iid (independent and identically distributed) inherent in datasets used for federated applications, offering a device-independent API; (ii) enables self-aware data security by attaching access policy to data and extending web access control specification; (iii) fosters open source serverless platforms to support non-functional requirements (NFRs), such as predictability, stateful computation and composability; (iv) boosts the serverless concept as a way to abstract functionalities deployed over MCUs on open-source serverless platform.

Finally, at the *decentralized intelligence level*, the meta-OS reference framework manages to: (i) support existing SOTA techniques in deep neural network (DNN) compression, reduction and optimization methods, while minimally affecting the performance of the models [54,55]; (ii) promote novel over-the-air transmission techniques for FL and transfer learning to effectively reduce the communication and energy expenditure [56,57,58,59]; (iii) incorporate open source MLOps toolchains to address current lacking areas in supporting the decentralized intelligence concept, such as in the model development, deployment and operations domain.

## 5. Potential Applications in Diverse Domains

**Figure 4:**
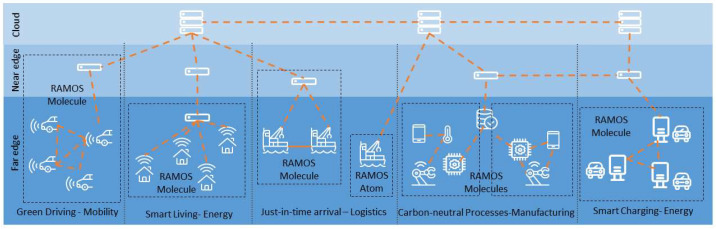
RAMOS components in diverse domain scenarios, illustrating the different orchestration, communication and data sharing patterns.

A meta-OS platform based on the proposed RAMOS architecture can be used in different domains, particularly in those demanding data-intensive edge intelligence and the deployment of distributed applications across multiple organizations and geographies. These domains may cover various perspectives of modern life, including green driving, port logistics and transportation, smart living, carbon-neutral manufacturing and use of renewable energy sources (conceptualized in Figure 4), to name a few. Applications discussed in the above domains require dynamic orchestration, communication and data exchange patterns, characteristics that are provisioned in the RAMOS architecture. Next, we introduce some potential applicability scenarios, describing the current practices and then highlighting the potential benefits to be achieved by leveraging the RAMOS solution.

To concretely describe how AI/ML applications can be supported within RAMOS architecture, five realistic use case scenarios are outlined. The *training of an AI/ML* optimization model is the process of properly adjusting the model weights so as to ensure minimization of an error/loss function. During this process, the training data could be gathered either in a centralized or an edge location. *Inference of an AI/ML model* refers to the usage of the model for making predictions or providing alarms, without modifying its parameters/weights. Regarding the AI/ML-related blocks of RAMOS, the general training and inference concept proposed for all the presented scenarios is in line with the following pattern: each Atom/device (i.e., the OBU hardware in car, the energy meter in the house, etc.) runs a trained (regression/classification) model that has been initially trained with either local data or data exchanged across Atoms in a peer-to-peer fashion. If needed, the Atoms exchange model parameters (instead of raw data that would jeopardize security and privacy) with Molecules, so that a more accurate model can be trained. This new model is then fed back to the Atoms for a more optimized inference.

### 5.1. Green Driving for Reduced Fuel Comsumption and Decreased Vehicle Emissions

The automotive industry is leading the global activities towards setting the bases for the future autonomous vehicle. The ultimate goal is to create smart and interactive cars that will continuously learn from different situations happening on the road and provide vehicle-to-everything (V2X) services, including the optimization of driving patterns of citizens. In the current setting, cars are equipped with an on-board unit (OBU) connected to the on-board diagnostics (OBD) system, which is an automotive embedded system that gathers data [60,61]. This data, along with notifications for anomaly occurrences (for instance, ice in the street, accident, break down, etc.), can be sent and received by vehicles, pedestrians and infrastructure (e.g., traffic lights).

Currently, most of the vehicles collect information, such as the instant consumption of gas/petrol, and they provide recommendations to drivers to reduce energy consumption, in the form of messages on screen or, in a few cases, hydraulic pressure on the gas pedal. However, these recommendations are based on suboptimal decisions, as they solely rely on a static and predefined model installed on the vehicle that is based on static rules, taking into consideration data only from a single vehicle [62,63]. Even in the case of V2X communication, where vehicles exchange information for the purpose of early warning signals (e.g., accident in front), data processing takes place in the cloud (the so-called multi-access edge computing or MEC), increasing latency far beyond those limits that may drive an undesired risk of late critical reactions.

**Figure 5:**
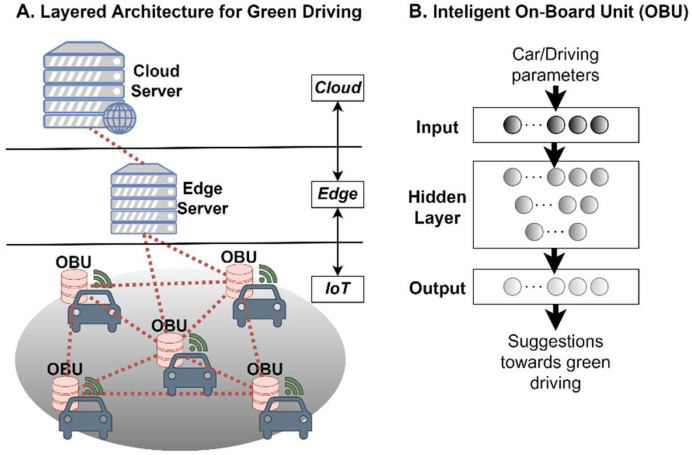
Resources and model sharing in the RAMOS architecture amongst the individual vehicles’ OBUs in the green driving scenario.

By leveraging the proposed RAMOS platform, the following paradigm shifts emerge: (i) the model of each individual vehicle will be derived using ML-based approaches, enabling the continuous and dynamic model update once new data arrive, in contrast to static rule-based models that are currently used; (ii) the OBU devices will be able to create ad-hoc cloud networks (e.g., in traffic lights or on the move) and share data and models in a secure manner, following the peer-to-peer continuum concept, contradicting the current scheme where any type of exchange between vehicles is performed through MEC; (iii) under the orchestration capabilities of the OBUs (acting as Atoms) and the nearby base station (acting as a Molecule), the resources of the vehicles can be shared and dynamically allocated for ML training or inference purposes, as opposed to the current hierarchical continuum scheme that lacks coordination of dynamic resources, as depicted in Figure 5; (iv) under the concept of decentralized intelligence, each individual ML model will be based on the collective knowledge of multiple cars and driver attitudes, making it more accurate and providing it with better generalization capabilities; (v) the ML training convergence time is reduced due to sharing pre-trained models among vehicles, as well as due to the ability of sharing computational resources among Atoms belonging to the same ad-hoc cloud network. By adopting the aforementioned technological paradigm shifts, valuable recommendations regarding proactive warnings in the case of car accidents as well as optimal speed and acceleration/deceleration of the vehicle may be offered to the drivers via text or voice messages. In this context, each car’s OBU will have its individual ML model that has been trained with the vehicle’s own collected data. Since the performance of each model depends on the amount of available data (cars that have travelled numerous km are expected to have better trained models), two or more vehicles will be able to ad-hoc exchange their model parameters to increase the accuracy of their predictions, or even share resources for computationally intensive training tasks (for instance when cars are immobilized due to traffic conditions).

### 5.2. Smart Living for Migration to Preferable Energy Consumption Behaviour and Predictive Maintenance of Electrical Equipment

Electric meters are installed by utility companies at households to survey the usage of electric power in residential settings, while also providing citizens access to their daily energy consumption and other limited information, making them aware of their charges and habits. However, a more fine-grained consumption profile can be created based on a novel technology that is under research during the last few years, called non-intrusive load monitoring (NILM) [64,65,66]. NILM can provide information on energy consumption per appliance type, such as electric lamps, washing machines or television, based on time variations of voltage, current, power factor and other variables collected at a sub-minute rate. Such a consumption profile would be much more useful, both to utility companies to accurately predict future energy demands and enable green energy sources, but also for consumers interested in changing habits and reducing their energy footprint.

However, the current approach faces the following drawbacks: First, the data extracted by the NILM is processed in the cloud and is mostly beneficial for the utility companies (e.g., for the purpose of accurate energy demand prediction). Second, recommendations towards the consumers or even direct corrective actions (e.g., actuator that is instructed to turn off the lights) rely solely on the individual datasets of each consumer, restricting further knowledge and thus decision optimality. Third, there are strong concerns for privacy, and, in general, on the data governance model that is applied to the consumers’ data (collected from the smart meters installed today).

**Figure 6:**
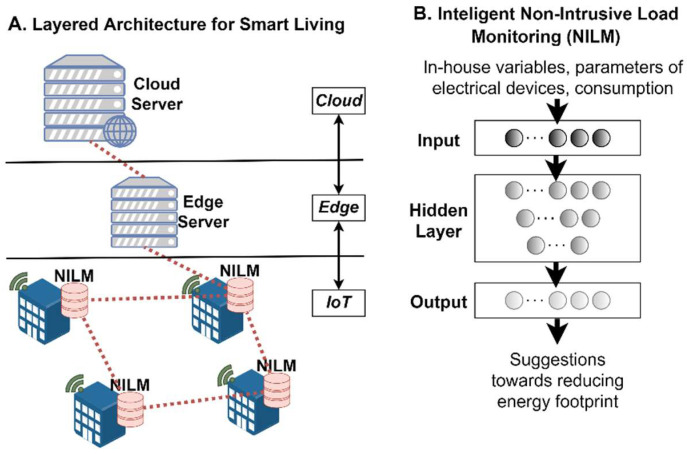
The interconnection of NILM ML model and data sharing in the RAMOS framework, targeting to provide suggestions towards energy footprint reduction at households.

By adopting the RAMOS architectural framework, as highlighted in Figure 6, the following advancements can be reached: (i) enhancement of the computational capacity on the NILM device (far edge) in order to minimize data traffic and data processing at cloud, overcoming current centralized data processing schemes; (ii) exploitation of novel privacy-preserving algorithms by sharing only parameters of the trained distributed models, contrary to sharing sensitive consumer data with external entities in a hierarchical manner; (iii) application of SL training algorithms at the edge (NILM devices/Atoms) that will improve the accuracy of the ML models, since the training process can be based on enhanced datasets in contrast to home-based training approaches of today. These advancements will enable the identification of energy consumption patterns for each specific home, allowing an accurate behavioral pattern analysis. In this way, consumers can be supported to change consumption behavior, since personalized recommendations are provided based on the actual consumption patterns and the exchange of ML trained models. Additionally, these shifts may create added value in the form of low-cost predictive maintenance of malfunctioning equipment (e.g., increased consumption of a device during a period of time might reveal a device malfunction). Therefore, a local ML model integrated in the NILM device that has been trained utilizing the collective energy consumption datasets of several households (i.e., models generated from houses in the same geographic area or even from houses with similar energy consumption profiles) can provide suggestions to improve the users’ behavioral energy habits (e.g., stop regulating the heating/cooling of the room temperature) or warnings in the case that an appliance is deviating from its usual performance (e.g., electrical leakage).

### 5.3. Just-in-Time Arrival for Vessel Traffic Management and Port Logistics

Although maritime transport and ports logistics play a key role in trading, these activities significantly contribute to the greenhouse gas (GHG) emissions [67]. The ports, as intermodal hubs, play a pivotal role in the transportation chain, as facilitators of the flow of people and cargo, but also as concentration points of emissions. Currently, the majority of port services are delivered ad-hoc as requested by each vessel and based on availability of resources, without any consideration on optimization of berth and crane allocation, or transportation equipment usage to/from the storage area [68].

The shortcomings of the current solutions include: (i) port services (availability of dock, availability of crane, berth, etc.) are provided in a more or less uncoordinated manner, resulting in extended delays on port service provisioning, leading to inefficient freight and passenger flows; (ii) estimated time of arrival of the vessels is partially taken into consideration on port planning, thus introducing extended time that vessels are waiting outside the port, resulting in increased fuel consumption and GHG emissions; (iii) data regarding vessel operational parameters (speed, power, fuel consumption, etc.) that are locally collected, are only used for monitoring purposes and neither for optimizing the time of arrival of the ship to the port, nor for optimization of its route with respect to energy savings.

**Figure 7:**
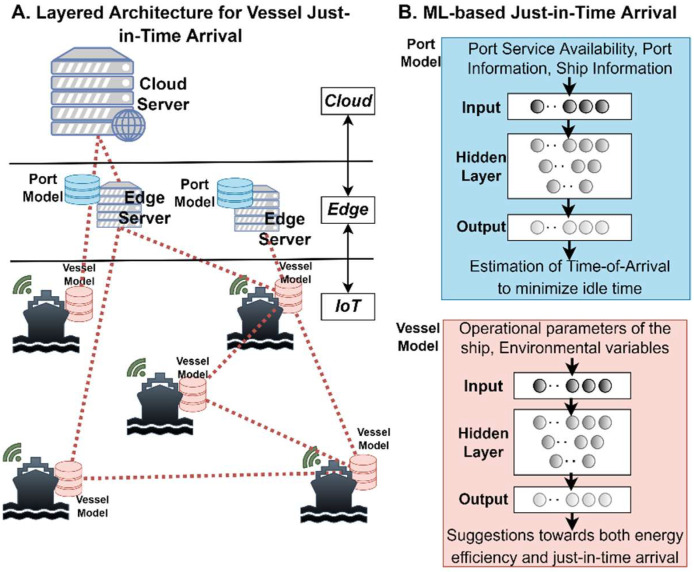
Centralized ML port model and distributed ML vessel models coordinating in the RAMOS platform towards increasing the port efficiency and port call optimization, while also reducing greenhouse gas emissions.

The meta-OS platform based on the proposed RAMOS architecture can lead to the following evolutions: (i) the port informs each vessel for the optimally scheduled time of arrival based on port service availability, thus minimizing the vessel idle time outside the port, contradicting current practices; (ii) based on the port schedule, the vessel uses its individual computational resources (Atom) to train an ML model based on the operational parameters and combined with environmental ones (wind speed, direction, waves, etc.) to optimize its voyage planning (routing, vessel speed) to the port, respecting energy efficiency aspects in contrast to using data only for monitoring purposes, as illustrated in Figure 7; (iii) the information gathered at the management platform of the port (RAMOS Molecule) will be continuously enriched with data regarding the accurate and efficient delivery of port services to the vessels, creating a labeled dataset that can be exploited for further training of the port ML algorithm for optimized port service scheduling. In this framework, a distributed ML model can be integrated in the hardware of each vessel (continuously trained with its individual operational data) and can provide recommendations for the optimized ship’s speed and route planning, taking into account the time of arrival (as scheduled by the port based on the availability of its services) and the environmental weather conditions. Moreover, a centralized ML algorithm deployed in the port (Figure 7), will indicate the optimized scheduling for just-in-time arrival provision of port services. The platform solutions will provide a means of increasing port efficiency and port call optimization which significantly contributes to reduced GHG emissions as a result of the ship’s speed to arrive just in time. This also reduces anchorage time and congestion in the port area, supports better orchestration of berth, crane and storage allocation and enables efficient freight and passenger flows.

### 5.4. Energy Reduction towards Carbon-Neutral Manufacturing Processes

Energy consumption reduction at the regional level is the main priority in the manufacturing domain [69,70]. Manufacturing companies have already deployed zero net emission programs to respond to climate change challenges [71]. In this context, companies with factories in different regions use energy management software mainly to track and monitor each factory consumption and to take decisions on potential improvement at local level.

In the current situation, each factory energy manager uses the monitoring system to only improve the local state, based on their own datasets, thus increasing the time of the training process and diminishing the accuracy of the non-ML-based model. Furthermore, the datasets are usually in a non-standardized format even among the various processes of the same factory, resulting in time-consuming data analysis. Finally, data coming from different factories are centrally gathered for cross-elaboration, but this process requires additional data normalization and standardization procedures to enable best cross fertilization practices among the factories.

**Figure 8:**
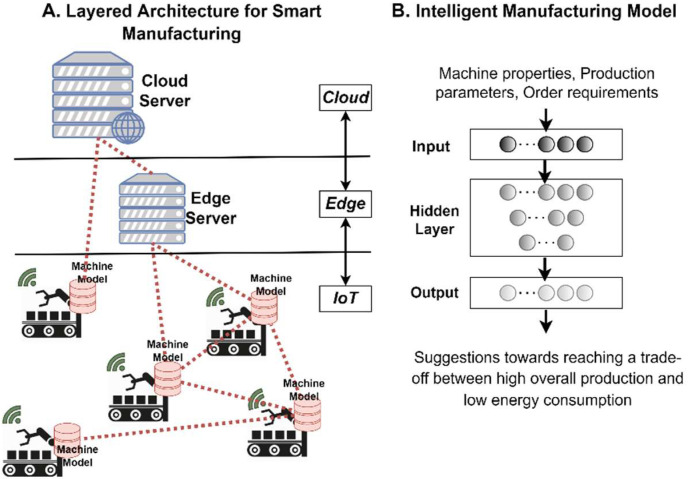
Implementation of the RAMOS layered architecture in data and ML model sharing amongst several factory sites towards carbon-neutral manufacturing.

Leveraging a meta-OS platform in accordance with RAMOS principles, the following advancements in the manufacturing sector unfold: (i) at a local level, ML algorithms can be trained targeting energy consumption reduction, based on factory-specific available datasets, supporting the decision making process in terms of resource and process optimization, as well as cost reduction initiatives, as opposed to the rule-based, static models that are currently used (Figure 8); (ii) supporting heterogeneity of devices, as well as standardized approaches on data format and modeling, the meta-OS can considerably reduce the time required for data pre-processing in comparison to extended time-consuming and error-prone data analysis performed nowadays; (iii) early adoption of already trained ML models by a newly established factory site is enabled, based on model sharing techniques (e.g., an already trained model can effectively minimize the time needed for training a new model based on local data of the newly established factory). By adopting the proposed platform and technological solutions, the impact on business involves the reduction of consumed energy related to the overall production. Towards this direction, the energy reduction-targeted ML model of each factory will be continuously trained, not only by utilizing its own dataset from the production line, but also by using cooperative intelligence that is shared among the factory sites with reduced communication overhead. In addition, datasets that are generated by heterogeneous devices will be homogenized and effortlessly employed for training or post-processing purposes.

### 5.5. Smart Charging Stations for Electric Vehicles

**Figure 9:**
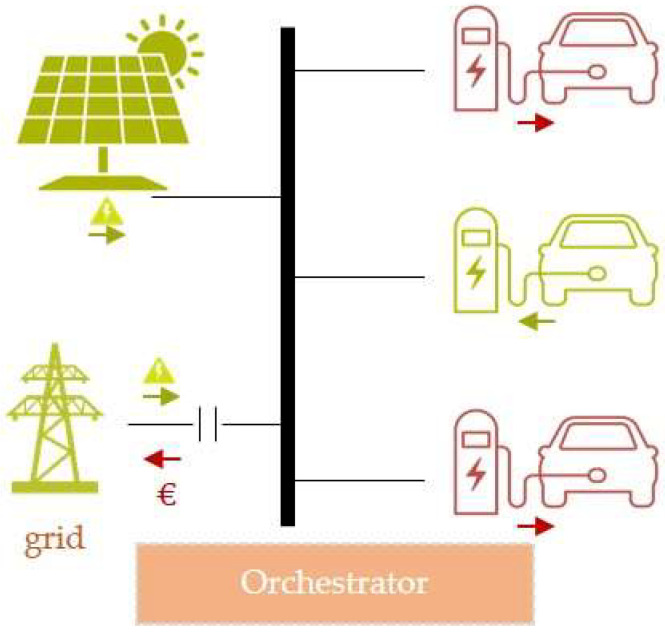
High-level overview of an EV charging system, where RES are integrated with the conventional power grid.

The majority of distribution system operators (DSOs) implement an electric vehicle (EV) charging system, where renewable energy sources (RES) are integrated with conventional grid power sources [72], as depicted in Figure 9. The EV chargers work as load to the electric system (depicted in red), while vehicle-to-grid (V2G) bi-directional chargers can work both as load and source of stored energy to the system (depicted in green). The RES energy is directly injected into the grid and the EV chargers start working as soon as they have an EV connected. Nowadays, EV charging premises use a simple management solution: if there is an EV connected, the system charges the car; if not, the EV charger is in a standby mode [73].

The current implementation of an EV charging ecosystem includes the following drawbacks: (i) the data collected from EV charging are mainly used for monitoring purposes and not for optimization, neither with respect to the charging hour nor considering the increased use of green energy coming from RES; (ii) the management of such a system is centralized in nature and disregards substantial functionalities or algorithms concerning resource optimization; (iii) the existing system does not support mechanisms for fault tolerance and, thus, it is prone to failure if an EV charger node stops sending data.

The RAMOS architecture can be adopted by the EV charging scenario, bringing the following benefits: (i) provision of ML-assisted decisions to optimize the energy transfer function (generation/storage/load) and the charging hour, so that the cheapest energy schedule or the hours with the maximum RES generation are exploited, in contrast to current solutions that lack any optimization process; (ii) orchestration of the computational resources at the charging station (Molecule running local ML on the edge) to acquire data from loads, storage and RES, while at the same time balancing with the energy coming from the grid; (iii) adoption of edge data management to reduce data communication overhead between EV chargers and the EV charging grid central management solution; (iv) implementation of federated/swarm intelligence techniques among the edge nodes, bringing a fine-tuned and customized charging experience at a local level and rewarding the users for letting the chargers decide when to proceed with charging in a predefined time frame. Moreover, secondary charging equipment can store the monitoring data (energy supplied, time of charging, battery levels, end-user id, green energy sources). Therefore, the ML model that will be integrated in the hardware of an EV charger will provide suggestions on the charging procedure of each user, based on both his/her charging profile, the traffic distribution of the users requesting charging services and the availability of green renewable energy. The training of each ML model will consider the collaborative knowledge extracted by all EV chargers through model sharing, minimizing at the same time the amount of data transferred via the network.

## 6. Business and Societal Impacts

The implementation of the proposed meta-OS reference architecture, that notably advances current cloud–edge–IoT architectures, essentially supports the development of decentralized intelligent applications, while at the same time reducing the complexity of managing distributed architectures and the associated operational cost of cloud–edge architectures. Moreover, RAMOS promotes an open source-based digital infrastructure to create data spaces following a peer-to-peer and trust-centric approach that transforms the existing balance between cloud and edge workload by enabling data processing in smart connected objects at the edge and leads to enhanced energy efficiency. The above points are further discussed in the following subsections.

### 6.1. Towards Data Sharing Principles and an Open Edge Ecosystem

Data nowadays play a central role in the development of innovative connected services and products. Contrary to the case of core clouds, however, there is not yet much investment on public edge infrastructure, thus limiting the available offer and imposing a CAPEX model, not ideal, especially for small and medium-sized enterprises (SMEs). SMEs can presently only benefit from their own limited datasets to develop innovative products and ML algorithms, which may not apply to other markets and paradigms. Furthermore, the rules for personal and industrial data exchange and processing are becoming stricter, and privacy preservation has become a central activity for enabling the implementation of any data-driven services [74]. In such a context, GDPR compliance and full transparency toward citizens/data subjects become additional requirements, which developers need to address from privacy by design to informational self-determination. RAMOS intrinsically targets at increased data sharing as a consequence of technologies that put data owner at the center, promoting trust over data control, and creating wealth and adding value to new innovative connected services and products, enabling an open ecosystem for ad-hoc dynamic IoT infrastructures. Furthermore, the proposed architectural framework supports the dynamic federation of edge resources, enabling the creation of an ecosystem of edge resource providers and nurturing novel business models and technical approaches to create innovative cloud–edge solutions, extending the involvement of SMEs in the data markets for sharing industrial data.

### 6.2. Towards Digital Transition for Clean Energy and Climate Net Neutrality

As highlighted in the present work, the proposed architecture fundamentally enables energy-aware multi-dimensional computational offloading, inherently contributing to climate neutrality (e.g., CO_2_ footprint minimization and increased use of renewable energy sources). To this end, energy-aware data infrastructures will avoid the explosion of ICT footprint and provide deeper understanding of decentralized intelligence to support green digital solutions as described in the potential application scenarios: (i) exploiting ML capabilities to process data from smart connected objects and decide the corrective actions in the energy grid sector, thus enabling efficient energy management in smart cities; (ii) focusing on the green driving principles by optimizing the speed/acceleration of vehicles in order to minimize the environmental footprint, identifying driving patterns and optimizing drivers’ behavior that will eventually lead to a reduction in car pollutant gases and fuel saving; (iii) promoting advancements in the IoT technologies of the manufacturing domain in the framework of Industry 4.0 by providing AI-assisted distributed solutions in predictive maintenance and quality management, as well as boosting productivity, improving safety and reducing the environmental footprint of the manufacturing sector; (iv) providing a unified port-related management system for key logistics operations such as traffic management, allocated port resources and waste monitoring, targeting to reduce the energy consumption and GHG emissions associated with the maritime transportation sector; (v) employing distributed intelligence principles to effectively use smart EV charging from renewable sources at the edge.

Towards this direction, companies and public organizations that are active in domains requiring real-time decision-making can adopt the proposed architecture for reduction of their energy footprint. In this way, RAMOS has an indirect impact on the quality of life of citizens, since it results in energy consumption reduction associated with their homes, minimization of car pollutant gases’ emission, fuel saving and, finally, more efficient passenger flow in transportation.

## 7. Conclusions

In the present work, we propose a reference architecture of a meta-OS (RAMOS) that aims to transform current vertically-siloed cloud–edge-IoT architectures in a dynamic, distributed and trusted continuum. The presented architecture advances current technical solutions by transposing: (i) from a hierarchical continuum to a peer-to-peer continuum; (ii) from the orchestration of static resources to the coordination of dynamic resources; (iii) from service-gravity to data-gravity; (iv) from central learning to swarm learning methods; (v) from data-aware to context-aware machine learning operations (MLOps). This novel architectural approach will be capable of hosting next-generation ML-based and data-intensive applications at the edge, taking into account their diverse requirements. The specific layered architectural components of the RAMOS agents (Atoms, mainly for embedded devices and Molecules for servers and clouds) and their functionalities are fully described, along with their interconnection and coordination principles in a secure and trusted manner.

The potential implementation of the proposed architectural framework is then highlighted in industrial and business sectors that require real-time decisions based on huge amounts of received information and data. In these scenarios, we demonstrate RAMOS distributed intelligence and privacy preservation principles used for data sharing purposes in use cases that do not follow the same communication and orchestration patterns. In addition, we cover issues related to the reduction of the energy consumption in domains with diverse characteristics, ranging from the green driving with ultra-low latency requirements to the smart living, aiming at household-level energy efficient enhancement. Furthermore, the direct and wider impacts emanating from the adoption of the proposed architectural approach are presented.

Finally, our future work includes the implementation and validation of the domain-agnostic characteristics of the proposed meta-OS architecture in the described business sectors and illustrate how RAMOS can affect real-time decision making in sensitive domains, also quantifying the efficiency of the current architectural scheme. Moreover, our future research directions involve the investigation of ML techniques that can truly enable distributed learning at the (far) edge by become context-aware, rather than just being data-aware, i.e., by dynamically adapting the ML lifecycle to the context (i.e., data localization, energy).
